# Supplementary material for: Identification of photoperiod-induced specific miRNAs in the adrenal glands of Sunite sheep (Ovis aries)
Source: Front Vet Sci. 2022 Jul 22;9:888207. doi: 10.3389/fvets.2022.888207 (PMC9354845; doi:10.3389/fvets.2022.888207)
Supplement: Supplementary file 1 [file Data_Sheet_1.ZIP › Supplementary file/miRDeep2 file.zip!/miRDeep2 file/Novel_Structure/LP42A3/Novel_85.pdf]

Provisional ID	:	NC_019467.2_12206
Score total	:	2.4
Score for star read(s)	:	-1.3
Score for read counts	:	0
Score for mfe	:	2.1
Score for randfold	:	1.6
Score for cons. seed	:	
Total read count	:	132
Mature read count	:	132
Loop read count	:	0
Star read count	:	0

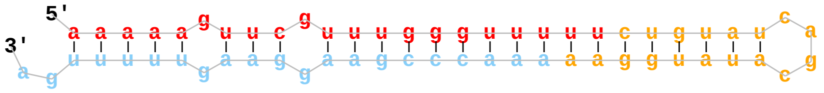

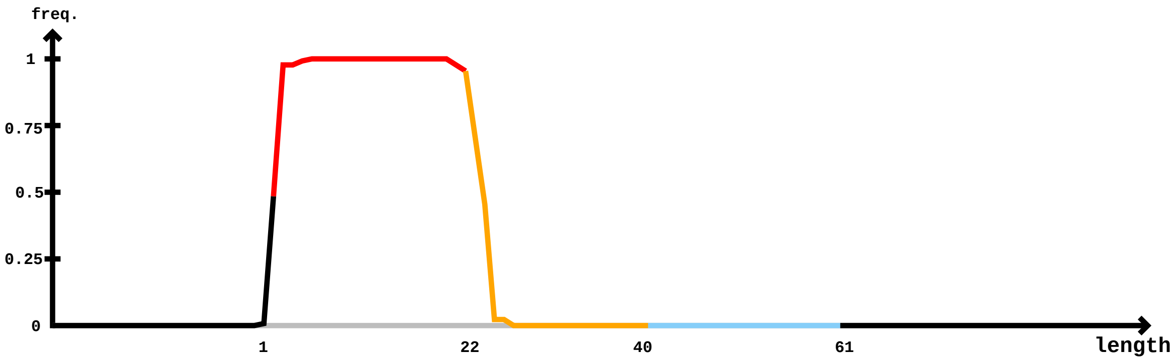

Mature **Star**

```
5'- gcaucauauauuggguuggcgaaaaaguucguuuggguuuuucuguaucagcauauggaaaaacccgaaggaaguuuuugaacaacccaauaugauauguucaaaauggaug   -3'    exp
    ((((..((((((((((((...(((((.(((.((((((((((((((((((....))))))))))))))))))).))).)))))...))))))))))))..))))...........   reads       mm          sample
    ....................gaaaaaguucguuuggguuuuu......................................................................   1           0           seq
    .....................aaaaaguucguuuggguuu........................................................................   3           0           seq
    .....................aaaaaguucguuCggguuuu.......................................................................   1           1           seq
    .....................aaaaaguucguuugUguuuuu......................................................................   1           1           seq
    .....................aaaaaguucguuuggguuuuu......................................................................   52          0           seq
    .....................aaaaaguucguuuggguuuCuc.....................................................................   1           1           seq
    .....................aaaaaguucguuuggguuCuuc.....................................................................   1           1           seq
    .....................aaaaaguucguuuggguuuuucA....................................................................   4           1           seq
    ......................aaaaguucguuuggguuuC.......................................................................   2           1           seq
    ......................aaaaguucguuuggguuuuuU.....................................................................   10          1           seq
    ......................aaaaguucguuCggguuuuucu....................................................................   3           1           seq
    ......................aaaaguucguuuggguuuuucG....................................................................   2           1           seq
    ......................aaaaguucguuuggguuuuucu....................................................................   7           0           seq
    ......................aaaaguucguuuggguuuuucA....................................................................   41          1           seq
    ........................aaguucguuuggguuuuCcugu..................................................................   2           1           seq
    .........................aguucguuuggguuuuCcugu..................................................................   1           1           seq
```
